# Individual and joint impact of family history and *Helicobacter pylori* infection on the risk of stomach cancer: a nested case–control study

**DOI:** 10.1038/sj.bjc.6602067

**Published:** 2004-07-27

**Authors:** H Yatsuya, H Toyoshima, A Tamakoshi, S Kikuchi, K Tamakoshi, T Kondo, T Mizoue, N Tokui, Y Hoshiyama, K Sakata, N Hayakawa, T Yoshimura

**Affiliations:** 1 Department of Public Health/Health Information Dynamics, Field of Social Life Science, Program in Health and Community Medicine, Nagoya University Graduate School of Medicine, 65 Tsurumai-cho, Showa-ku, Nagoya, Aichi 466-8550, Japan; 2 Department of Preventive Medicine/Biostatistics and Medical Decision Making, Field of Social Life Science, Program in Health and Community Medicine, Nagoya University Graduate School of Medicine, Nagoya, Japan; 3 Department of Public Health, Aichi Medical University, Aichi, Japan; 4 Department of Medical Technology, Nagoya University School of Health Sciences, Nagoya, Japan; 5 Department of Preventive Medicine, Kyushu University Graduate School of Medical Sciences, Fukuoka, Japan; 6 Department of Clinical Epidemiology, Institute of Industrial Ecological Sciences, University of Occupational and Environmental Health, Kirakyushu, Japan; 7 Department of Public Health, Showa University School of Medicine, Tokyo, Japan; 8 Department of Public Health, Wakayama Medical University, Wakayama, Japan; 9 Department of Epidemiology, Hiroshima University Research Institute for Radiation Biology and Medicine, Hiroshima, Japan

**Keywords:** family history, *Helicobacter pylori*, stomach cancer, sex difference, nested case–control study, JACC study

## Abstract

We used 202 cases of stomach cancer and 394 controls nested within the Japan Collaborative Cohort Study For Evaluation of Cancer Risk (JACC study) to investigate whether family history has an independent effect on the risk of stomach cancer after controlling for the *Helicobacter pylori* infection. A positive history of stomach cancer in one or more first-degree relatives was associated with an increased risk of the disease in women, but not in men after controlling for *H. pylori* infection and other confounding variables. Women with both a family history and *H. pylori* infection were associated with more than five-fold increased risk of the disease (OR 5.10, 95% CI 1.58–16.5) compared to those without these factors. These results suggest the existence of inherited susceptibility to the disease in women, and that measurements of *H. pylori* infection together with the family history allow meaningful evaluation of risk beyond that provided by either factor alone.

Familial aggregation of stomach cancer has long been noted (Macklin, 1960; Toyoshima *et al*, 1997; Yatsuya *et al*, 2002; Kondo *et al*, 2003). Certain evidence, however, suggests that both genetic and environmental factors are responsible for familial clustering (Palli *et al*, 2001). One environmental risk factor is infection with *Helicobacter pylori* (*H. pylori*) (Talley *et al*, 1991), and previous studies have revealed that this also aggregates within families (Dominici *et al*, 1999).

A case–control study found that a family history of stomach cancer significantly increased the risk of the disease independent of *H. pylori* infection (Brenner *et al*, 2000). In addition, positive family history in individuals with *H. pylori* infection appeared to be a stronger risk factor for the disease compared to those without such an infection. There are, however, no prospective studies of this subject. We, therefore, conducted a nested case–control investigation within a cohort study to examine the independent effect of family history on the risk of stomach cancer after controlling first for the *H. pylori* infection, and, second, to evaluate any joint contribution of these two factors to the disease risk.

## MATERIALS AND METHODS

### JACC study

The study was part of the Japan Collaborative Cohort Study For Evaluation of Cancer Risk Sponsored by the Ministry of Education, Science, Sports and Culture of Japan (JACC Study), a nation-wide multicenter collaborative study to evaluate prospectively various risks or protective factors on cancer mortality and incidence. Details of the study design were reported previously (Ohno and Tamakoshi, 2001; Hoshiyama *et al*, 2002). Briefly, the cohort included 110 792 men and women (46 465 and 64 327, respectively), 40–79 years old at recruitment, enrolled in 1988–1990. Enrollment was based on the participants of a general health checkup that is periodically provided by the 45 municipalities involved. Informed consent procedures were approved by the Ethics Committee of Medical Care and Research, University of Occupational and Environmental Health, Kitakyushu, where the chief investigator of stomach cancer group is affiliated, and the Ethical Board of the Nagoya University School of Medicine, Japan, where the present chairman of the JACC study is affiliated.

At the time of recruitment, baseline characteristics were gathered by self-administered questionnaires, which covered the medical history and included lifestyle-related items such as drinking and smoking, level of education, and family history of several medical conditions including cancer. About one-third of the cohort members (*n*=39 293) also donated a residual serum sample (about 2 ml) used for the general health checkup. It was partitioned into 0.3–0.5 ml aliquots and stored at −80°C until laboratory analyses.

### Follow-up and identification of stomach cancer cases, and selection of control subjects

Vital statuses of the participants were checked annually by each regional research centre, with permission to review their population-register sheets from the Ministry of Public Management, Home Affairs, Post and Telecommunications. Incidence of cancer was ascertained in 24 study areas (*n*=65 184) and coded according to the tenth revision of International Classification of Diseases and the second edition of International Classification of Diseases for Oncology. These data were collected at the central office of the Research Committee.

We first restricted the subjects to those who lived in the study areas where cancer incidence was ascertained. We then excluded 857 participants with a self-reported history of cancer at any site. Among the remaining 64 327 subjects, diagnosis of stomach cancer 12 or more months after cohort recruitment was documented in 804 cases until the end of 1997. Serum had been obtained from 218 out of the initial 804 cases. However, seven cases without enough serum for laboratory analysis, and one case without an eligible control subject were excluded. In addition, one of the 24 study areas where family history was not assessed was excluded from the analysis. Thus, the study reported here included 202 cases (105 men and 97 women) in total. Lag time between blood sampling and stomach cancer diagnosis varied between 12 and 113 months (median 50 months). Each of these subjects was matched with two control subjects for gender, age at recruitment (as near as possible), and study area, who had also provided an adequate baseline blood sample and who were alive and remained free of confirmed cancer as of the end of 1997. Owing to a lack of eligible subjects, a few sets (*n*=10) contained only one control; a total of 394 controls was available for the present analysis. As information on the location of cancer within the stomach or the histological type was not available in all cases, we did not use it to classify cases.

### Laboratory assays

Serum samples from each case and matched controls were retrieved from storage and shipped on dry ice to a single laboratory for the assay. None of the samples had been previously defrosted. *H. pylori* infection was investigated serologically using HM-CAPTM (Enteric Products, Westbury, NY, USA) with antigen from Japanese (J-HM-CAP), and the serum titer of immunoglobulin G antibodies 2.3 or greater was defined as positive infection.

### Definition of family history and covariates

Family history of stomach cancer was defined as when the subject had at least one first-degree relative with a history of stomach cancer. Risk factors that could potentially confound the relation between family history and stomach cancer other than *H. pylori* infection were collected at the baseline, using a self-administered questionnaire. A drinking habit was first categorised into three statuses (none, past, present). If present, it was further categorised into two levels by weekly consumption (light, heavy), that is, daily alcohol consumption times days of drinking per week. Smoking status was classified into three levels (never, past, current). Consumption frequency of vegetables, citrus fruits, and green tea was initially assessed in five levels (everyday, 3–4 times a week, 1–2 times a week, 1–2 times a month and seldom). For the present analysis, the former two and the latter three categories were combined. Salty-food preference was categorised into three levels (dislike, neutral, like). Information on educational level was measured as the age of formal schooling completed and was classified into two categories: ⩽15 years old (corresponds to ⩽9 years of schooling) and ⩾16 years old (corresponds to ⩾10 years of schooling). Missing values in each variable were treated as an additional category in the variable, and were included in the analyses.

### Statistical analysis

We compared the baseline characteristics of case subjects and control subjects by one-way analysis of variance for continuous variables and *χ*^2^ tests for categorical variables. We then performed logistic regression analysis, conditioned on the matching variables of gender, age, and study area, and presented the odds ratios (ORs) that represent the risk associated with a positive family history of stomach cancer. Adjusted estimates of risk were obtained with multivariate models that controlled for *H. pylori* infection, and other covariates listed above. To assess the joint effect of family history and *H. pylori* infection on the risk of stomach cancer, four categories were created by the combination of these two factors. Another logistic regression analysis was performed taking a category with no family history and no *H. pylori* infection as a reference. The 95% confidence intervals (95% CIs) are presented for all ORs. All reported *P*-values are two-sided. All analyses were performed separately for men and women with SPSS statistical package for windows version 11.5.

## RESULTS

Table 1

**Table 1:** Baseline characteristics of the study participants

	**Men (*n*=307)**			**Women (*n*=289)**		
	**Cases**	**Controls**	***P*-value**	**Cases**	**Controls**	***P*-value**
**Characteristic**	**(*n*=105)**	**(*n*=202)**		**(*n*=97)**	**(*n*=192)**	
*Age category: no.* (%)						
40–49	6 (5.7)	12 (5.9)	Matching factor	9 (9.3)	18 (9.4)	Matching factor
50–59	22 (21.0)	42 (20.8)		32 (33.0)	64 (33.3)	
60–69	52 (49.5)	104 (51.5)		39 (40.2)	77 (40.1)	
70–79	25 (23.8)	44 (21.8)		17 (17.5)	33 (17.2)	
Age (years): mean±s.d.	63.6±7.9	63.3±7.8		61.6±8.3	61.4±8.3	
						
*H. pylori infection: no.* (%)						
Present	91 (86.7)	161 (79.7)	0.16	88 (90.7)	151 (78.6)	0.013
Absent	14 (13.3)	41 (20.3)		9 (9.3)	41 (21.4)	
						
*Family history of stomach cancer: no.* (%)						
Present	16 (15.2)	33 (16.3)	0.87	24 (24.7)	29 (15.1)	0.054
Absent	89 (84.8)	169 (83.7)		73 (75.3)	163 (84.9)	
						
*Number of siblings*						
0–2	11 (10.5)	28 (13.9)	0.73	13 (13.4)	25 (13.0)	0.97
3–5	46 (43.8)	79 (39.1)		42 (43.3)	88 (45.8)	
6–	31 (29.5)	57 (28.2)		28 (28.9)	51 (26.6)	
Missing	17 (16.2)	38 (18.8)		14 (14.4)	28 (14.6)	
						
*Smoking status: no.* (%)						
Never	17 (16.2)	35 (17.3)	0.35	86 (88.7)	168 (87.5)	0.52
Past	31 (29.5)	57 (28.2)		1 (1.0)	1 (0.5)	
Current	55 (52.4)	97 (48.0)		4 (4.1)	4 (2.1)	
Missing	2 (1.9)	13 (6.4)		6 (6.2)	19 (9.9)	
						
*Alcohol intake: no.* (%)						
None	21 (20.0)	42 (20.8)	0.41	69 (71.1)	146(76.0)	0.82
Past	7 (6.7)	6 (3.0)		3 (3.1)	4 (2.1)	
Light drinker	37 (35.2)	83 (41.1)		13 (13.4)	21 (10.9)	
Heavy drinker	23 (21.9)	34 (16.8)		0 (0.0)	1 (0.5)	
Missing	17 (16.2)	37 (18.3)		12 (12.4)	20 (10.4)	
						
*Educational level: no.* (%)						
⩽9 years of schooling	27 (25.7)	65 (32.2)	0.49	32 (33.0)	45 (23.4)	0.19
⩾10 years of schooling	56 (53.3)	100 (49.5)		46 (47.4)		
Missing	22 (21.0)	37 (18.3)		19 (19.6)	37 (19.3)	
						
*Salty-food preference: no.* (%)						
Dislike	10 (9.5)	31 (15.3)	0.57	24 (24.7)	29 (15.1)	0.11
Neutral	39 (37.1)	71 (35.1)		36 (37.1)	97 (50.5)	
Like	39 (37.1)	69 (34.2)		19 (19.6)	36 (18.8)	
Missing	17 (16.2)	31 (15.3)		18 (18.6)	30 (15.6)	
						
*Tomatoes: no.* (%)						
⩽1–2 times/week	52 (49.5)	105 (52.0)	0.89	47 (48.5)	94 (49.0)	0.61
⩾3–4 times/week	42 (40.0)	75 (37.1)		44 (45.4)	80 (41.7)	
Missing	11 (10.5)	22 (10.9)		6 (6.2)	18 (9.4)	
						
*Citrus fruits: no.* (%)						
⩽1–2 times/week	48 (45.7)	79 (39.1)	0.49	29 (29.9)	65 (33.9)	0.78
⩾3–4 times/week	44 (41.9)	91 (45.0)		56 (57.7)	106 (55.2)	
Missing	13 (12.4)	32 (15.8)		12 (12.4)	21 (10.9)	
						
*Spinach and green vegetables: no.* (%)						
⩽1–2 times/week	29 (27.6)	64 (31.7)	0.55	30 (30.9)	60 (31.3)	0.95
⩾3–4 times/week	65 (61.9)	112 (55.4)		58 (59.8)	112 (58.3)	
Missing	11 (10.5)	26 (12.9)		9 (9.3)	20 (10.4)	
						
*Carrots and pumpkins: no.* (%)						
⩽1–2 times/week	44 (41.9)	86 (42.6)	0.85	35 (36.1)	78 (40.6)	0.70
⩾3–4 times/week	52 (49.5)	95 (47.0)		52 (53.6)	98 (51.0)	
Missing	9 (8.6)	21 (10.4)		10 (10.3)	16 (8.3)	
						
*Green tea: no.* (%)						
⩽1–2 times/week	11 (10.5)	13 (6.4)	0.32	11 (11.3)	21 (10.9)	1.00
⩾3–4 times/week	93 (88.6)	184 (91.1)		83 (85.6)	165 (85.9)	
Missing	1 (1.0)	5 (2.5)		3 (3.1)	6 (3.1)	

shows the baseline characteristics of the 202 cases and the 394 matched controls. In this sample, the women diagnosed with stomach cancer were more likely to have a family history of stomach cancer, whereas men were not. The proportion of case subjects who reported a history of stomach cancer in a first-degree relative was 15.2 and 24.7% in men and women, respectively, against 16.3% in men and 15.1% in women in control subjects (*P*-values for the *χ*^2^ test were 0.87 in men, 0.054 in women; case *vs* control). The proportion of individuals infected with *H. pylori* was high even in control subjects (79.7 and 78.6% for men and women, respectively). However, it was higher in cases with stomach cancer in men and especially in women. Cases and controls did not differ significantly in terms of smoking status, alcohol intake, or other diet-related items for both sexes. The proportion of women with a higher educational level seemed to be higher in controls compared to that in cases.

Table 2

**Table 2:** Multivariate conditional logistic regression models examining the relation between family history and the risk of stomach cancer

	**Men (105 cases/202 controls)**			**Women (97 cases/192 controls)**		
**Variables adjusted for**	**RR**	**95% CI**	***P*-value**	**RR**	**95% CI**	***P*-value**
Univariate	0.96	0.48–1.91	0.907	1.92	1.02–3.64	0.044
Model 1	0.99	0.49–2.03	0.985	1.78	0.92–3.46	0.065
Model 2	0.89	0.40–1.97	0.768	1.73	0.82–3.65	0.153

CI=onfidence interval.

Model 1: Adjusted for *H. pylori* infection and the number of siblings (0–2, 3–5, 6+).

Model 2: Adjusted for *H. pylori* infection, the number of siblings (0–2, 3–5, 6+), smoking status (never, past, current), drinking habit self-rated preference of salty foods (dislike, neutral, like), consumption of green–yellow vegetables, citrus fruits and green tea (⩾3–4 times a week, ⩽1–2 times a week), and educational level (⩽9 years of schooling, ⩾10 years of schooling).

Missing values in each variable were treated as an additional category.

shows the relation of family history of stomach cancer to the risk of the disease incidence. Family history of stomach cancer was significantly related to the risk of the disease incidence only in women. This association became attenuated after adjustment for *H. pylori* infection or other potentially confounding variables. A family history of stomach cancer did not seem to be related to the risk of the disease incidence in men in the present dataset.

The prevalence of *H. pylori* infection in men with a family history was 81.6%, against 82.2% in men without such a history (Table 3

**Table 3:** Association between family history of stomach cancer and *H. pylori* infection, and joint contribution of family history and *H. pylori* infection on the risk of stomach cancer

		**No. of subjects**	***P* for 2 × 2 *χ*^2^ test**	**No. of cases**	**OR^a^**	**95% CI**	**OR^b^**	**95% CI**
*Men*								
*FH negative*	*Hp negative*	46 (17.8%)	1.00	12	1	(reference)	1	(reference)
	*Hp positive*	212 (82.2%)		77	1.63	0.80–3.33	1.81	0.79–4.15
*FH positive*	*Hp negative*	9 (18.4%)		2	0.76	0.13–4.44	0.72	0.11–4.86
	*Hp positive*	40 (81.6%)		14	1.64	0.62–4.32	1.66	0.54–5.12
								
*Women*								
*FH negative*	*Hp negative*	44 (18.6%)	0.234	7	1	(reference)	1	(reference)
	*Hp positive*	192 (81.4%)		66	2.92	1.22–6.99	2.98	1.10–8.02
*FH positive*	*Hp negative*	6 (11.3%)		2	2.02	0.30–13.6	1.84	0.17–19.9
	*Hp positive*	47 (88.7%)		22	5.30	1.87–15.0	5.10	1.58–16.5

CI=confidence interval; FH=family history; Hp=*H. pylori*.

a OR: Crude odds ratio.

b OR: Odds ratio adjusted for the number of siblings (0–2, 3–5, 6+), smoking status (never, past, current), drinking habit self-rated preference of salty foods (dislike, neutral, like), consumption of green–yellow vegetables, citrus fruits and green tea (⩾3–4 times a week, ⩽1–2 times a week), and educational level (⩽9 years of schooling, ⩾10 years of schooling).

Missing values in each variable were treated as an additional category.

). In women, the prevalence was 88.7 and 81.4% in those with and without a family history, respectively. The difference in the proportion was not significant, showing that a positive family history of stomach cancer and *H. pylori* infection were not related in this study sample, especially in men and to a lesser degree in women (*P*-value for *χ*^2^ test 1.00 and 0.23 for men and women, respectively).

In another logistic regression analysis comparing the risk of the disease among the four subgroups created by the combination of presence or absence of a family history and *H. pylori* infection, significantly increased risk (multivariate adjusted OR 5.10, 95% CI 1.58–16.5) was observed in women with a family history of stomach cancer and *H. pylori* infection compared with those without these risk factors. In men, however, no significant associations were observed.

## DISCUSSION

In this case–control study nested within a large-scale cohort of Japanese, we found that women with a family history of stomach cancer were associated with an increased risk of the disease independent of *H. pylori* infection. Women with both a family history and *H. pylori* infection had a greater than five-fold increased risk of the disease compared to those without these factors. The combined effect of these factors on the final risk of stomach cancer is approximately equivalent to the multiplicative product of the risks from the separate factors. Some biologic interaction between these two factors has been reported previously (Sepulveda *et al*, 2002). In a study of familial gastric cancer kindred, Rocco *et al* (2003) observed genetic abnormalities in the stomach of the first-degree relatives only in the presence of *H. pylori* infection, suggesting an interplay between the infection and the genetic profile of the host.

We did not find a significant association in men. This did not seem to be caused by a confounding of *H. pylori* infection. Some previous studies found stronger impact of family history on the disease risk in women than in men, which may partly be consistent with the present finding (Nagase *et al*, 1996; Yatsuya *et al*, 2002). Family history of stomach cancer was associated with a significantly increased risk of the disease (OR 4.5, 95% CI 1.3–15.2) in women, whereas it was related to a nonsignificant increased risk in men (OR 1.2, 95% CI 0.6–2.5) in a hospital-based case–control study in Japan (Nagase *et al*, 1996). The relative risk associated with a positive family history adjusted for age and the size of the family was 1.28 (95% CI 0.95–1.72) in men and 1.92 (95% CI 1.33–2.77) in women in a prospective study of Japanese (Yatsuya *et al*). However, other studies did not necessarily find the effect restricted to women (Palli *et al*, 1994; Inoue *et al*, 1998), which would suggest that the gender difference observed in the present study may be related to the study limitations.

First, the present study is based on about one-third of the cohort members who donated residual serum sample used for the general health checkup. Due to the fact that our previous study found an increased risk associated with a family history in men, though the increase was of borderline strength (Yatsuya *et al*, 2002), it might be possible that the male sample for this nested case–control study may potentially be biased. Future study with more cases with blood sample or with another indicator of *H. pylori* infection is needed to elucidate this issue.

Second, we did not classify cases by the location of cancer within the stomach or the histological type because the relevant information was not available in all cases. Stomach cancer in cardia was not associated with a family history of the disease in a case–control study conducted in Japan (Inoue *et al*, 1998). In addition, cancer in gastric cardia is reported to be associated more to environmental exposures, such as smoking or alcohol drinking (Inoue *et al*, 1994; Sasazuki *et al*, 2002), and environmental exposures in men were more diverse than in women, which may contribute to mask or exceed the effect of family history.

Third, recall of family cancer history is reported to differ between men and women, that is, women provided the history more accurately than men in a validation study (Kerber and Slattery, 1997); several studies have indicated the possibility of gender bias in recall as an explanation for the gender-specific association found in women (Wu *et al*, 1996). The lack of association in men in this study sample may be caused by a misclassification of subjects due to inaccurate reporting of family history, which would have attenuated the association.

Unexpectedly, family history of stomach cancer and serological prevalence of *H. pylori* infection assessed at the time of enrollment were not related in the combined sample of cases and controls in the present study. This may be due to a higher prevalence of *H. pylori* infection in the present sample than in the previous studies that found positive associations (45–70%) (Kikuchi *et al*, 1998; Brenner *et al*, 2000). Clearance of the infection in the course of development of stomach cancer via chronic atrophic gastritis may possibly explain the lack of association because such clearance is of likely relevance for some proportion of cases even when blood samples have been taken several years before diagnosis.

In conclusion, a family history of stomach cancer was associated with an increased risk of the disease in women. In addition, we observed that women with both a family history and *H. pylori* infection were associated with a greater than five-fold increased risk of the disease compared to those without these risk factors. Measurements of *H. pylori* infection together with the family history allow meaningful refinement of risk stratification beyond that provided by either factor alone. The study thereby partly confirms and extends the still quite limited empirical evidence on an issue that might well be relevant for potential screening strategies at least in women.

## Appendix A

### Japan Collaborative Cohort Study Group

The present members of the JACC Study and their affiliations are as follows: A Tamakoshi (present chairman of the study group, Nagoya University Graduate School of Medicine); M Mori (Sapporo Medical University School of Medicine); Y Motohashi (Akita University School of Medicine); I Tsuji (Tohoku University Graduate School of Medicine); Y Nakamura (Jichi Medical School); H Iso (Institute of Community Medicine, University of Tsukuba); H Mikami (Chiba Cancer Center); Y Inaba (Juntendo University School of Medicine); Y Hoshiyama (Showa University School of Medicine); H Suzuki (Niigata University Graduate School of Medical and Dental Sciences); H Shimizu (Gifu University School of Medicine); H Toyoshima (Nagoya University Graduate School of Medicine); S Tokudome (Nagoya City University Graduate School of Medicine); Y Ito (Fujita Health University School of Health Sciences); S Hashimoto (Fujita Health University School of Medicine); S Kikuchi (Aichi Medical University School of Medicine); A Koizumi (Graduate School of Medicine and Faculty of Medicine, Kyoto University); T Kawamura (Kyoto University Center for Student Health); Y Watanabe and T Miki (Kyoto Prefectural University of Medicine Graduate School of Medical Science); C Date (Faculty of Human Environmental Sciences, Mukogawa Women's University); K Sakata (Wakayama Medical University); T Nose (Tottori University Faculty of Medicine); N Hayakawa (Research Institute for Radiation Biology and Medicine, Hiroshima University); T Yoshimura (Institute of Industrial Ecological Sciences, University of Occupational and Environmental Health, Japan); K Fukuda (Kurume University School of Medicine); N Okamoto (Kanagawa Cancer Center); H Shio (Moriyama Municipal Hospital); Y Ohno (former chairman of the study group, Asahi Rosai Hospital); T Kitagawa (Cancer Institute of the Japanese Foundation for Cancer Research); T Kuroki (Gifu University); and K Tajima (Aichi Cancer Center Research Institute).
